# Stability and biological response of PEGylated gold nanoparticles

**DOI:** 10.1016/j.heliyon.2024.e30601

**Published:** 2024-05-01

**Authors:** Hoang Yen Nguyenova, Marie Hubalek Kalbacova, Marcela Dendisova, Miriama Sikorova, Jaroslava Jarolimkova, Zdenka Kolska, Lucie Ulrychova, Jan Weber, Alena Reznickova

**Affiliations:** a Department of Solid State Engineering, University of Chemistry and Technology Prague, 166 28, Prague, Czech Republic; b Institute of Pathological Physiology, 1st Faculty of Medicine, Charles University, 128 53, Prague, Czech Republic; c Faculty of Health Studies, Technical University of Liberec, Liberec, Czech Republic; d Department of Physical Chemistry, University of Chemistry and Technology Prague, 166 28, Prague, Czech Republic; e CENAB, Faculty of Science, J. E. Purkyne University, 400 96, Usti nad Labem, Czech Republic; f Institute of Organic Chemistry and Biochemistry of the Czech Academy of Science, 166 10, Prague, Czech Republic

**Keywords:** Nanoparticle, Gold, Polyethylene glycol, Stability, Cytotoxicity, Antiviral activity

## Abstract

Stability and cytotoxicity of PEGylated Au NPs is crucial for biomedical application. In this study, we have focused on thermal stability of PEGylated Au NPs at 4 and 37 °C and after sterilization in autoclave. Gold nanoparticles were prepared by direct sputtering of gold into PEG and PEG-NH_2_. Transmission electron microscopy revealed that NPs exhibit a spherical shape with average dimensions 3.8 nm for both AuNP_PEG and AuNP_PEG-NH_2_. The single LSPR band at wavelength of 509 nm also confirmed presence of spherical Au NPs in both cases. Moreover, according to UV–Vis spectra, the Au NPs were overall stable during aging or thermal stressing and even after sterilization in autoclave. Based on gel electrophoresis results, the higher density of functionalizing ligands and the higher stability is assumed on AuNP_PEG-NH_2_. Changes in concentration of gold did not occur after thermal stress or with aging. pH values have to be adjusted to be suitable for bioapplications – original pH values are either too alkaline (AuNP_PEG-NH_2_, pH 10) or too acidic (AuNP_PEG, pH 5). Cytotoxicity was tested on human osteoblasts and fibroblasts. Overall, both Au NPs have shown good cytocompatibility either freshly prepared or even after Au NPs′ sterilization in the autoclave. Prepared Au NP dispersions were also examined for their antiviral activity, however no significant effect was observed. We have synthesized highly stable, non-cytotoxic PEGylated Au NPs, which are ready for preclinical testing.

## Highlights

• PEGylated Au NPs were prepared by cathode sputtering into a liquid medium.
• Au NPs were stable during aging, after thermal stressing and even autoclaving.
• Au NPs were subjected to cytotoxic and antiviral testing.
• Au NPs displayed cytocompatibility with both fibroblasts and osteoblasts.
• Prepared Au NPs are safe for potential application in biomedicine.

## 1 Introduction

Noble metal nanoparticles (NPs) are one of the most studied nanomaterials with promising applications in many fields such as electronics, optoelectronics, catalysis and biology [1]. Among them, gold nanoparticles (Au NPs) are especially researched in biological applications due to their low toxicity, good cytocompatibility or ease of surface modification with peptides, DNA and antibodies. Above that, they also possess unique physico-chemical properties such as excellent absorbance and scattering of light. Based on these properties, Au NPs find use in biological diagnostics, cell labelling, medical imaging, and biological sensors [[2], [3], [4], [5], [6]]. Au NPs has shown potential in cancer therapy as drug carriers and recently as radiosensitizers in photothermal and photodynamic therapy [[5], [6], [7]]. Au NPs has also been exploited in antiviral therapy for their adjuvant effect for vaccines or as delivery systems of antiviral drug [8,9]. Some of the recent studies has also demonstrated Au NPs possessing antiviral properties against various viruses [7,10] (e.g. influenza A virus [11], herpes simplex virus [12,13], Zika virus [14], Dengue virus [15] or measles virus [16]).

Two major aspects, both related to the size of particular nanoclusters, responsible for NPs properties are large surface to volume ratio and the quantum size effect [17]. Therefore, the controlled and reproducible synthesis of Au NPs is necessary for their final applications [18].

Nanoparticles could be synthesized by either chemical or physical methods (physical vapour deposition, laser ablation, and *γ* radiation) [[19], [20], [21]]. Cathode sputtering is widely used and verified technique for metal nanostructures preparation that combines chemical and physical approach of NPs synthesis. The fundamental parameters for synthesis are type and properties of the liquid substrate. Metal sputtering onto specific liquid substrates creates dispersed metal nanostructures in the capturing media in single step [22,23]. Other advantage of this method consists of generation of clean NPs since neither chemical reactions nor additional stabilizing agents are required except the capturing media. Capturing media suitable for this synthesis are ionic liquids, vegetable oils or polymers e.g. polyethylene glycol [20,24].

Polyethylene glycol (PEG) is the most commonly applied non-ionic hydrophilic stealth polymer. PEG reduces the tendency of particles to aggregate by steric stabilization, thereby produced colloids have increased stability during storage and application [25]. PEG is cheap, variable, and on top of that, accepted by Food and Drug Administration for utilization in nanomedicine [26]. Moreover, it is easily accessible in various molecular weight or functionalized by different functional groups –amines, thiols to reactive groups, fluorescent colorants, vitamins, lipids or even enzymes [27]. Immobilization of Au NPs' surface with various polyethylene glycols (PEGylation) is one of the ubiquitously used procedures, which dramatically decreases nonspecific binding to cells and serum proteins. PEG coating decreases creation of a protein corona on NPs therefore the immune system does not identify them easily. The Au NPs’ circulation in body is consequently prolonged [28]. Diminution of nonspecific interactions of NPs inside organism via steric hindrance and control of surface charge distinctly reduce nanoparticle loss to improper place in the body, i.e., the bone marrow, spleen or liver [29].

Due to broad usage of Au NPs in bio-applications, it is crucial to answer the concern of their cytotoxicity. Bulk gold is considered inert and non-toxic [30,31]. However, there is a risk of Au NPs’ toxicity emergence due to their small size enabling them to penetrate and possibly negatively affect any cell in a body [32]. The toxicity of Au NPs is determined by their size, shape and surface charge etc. [33] Pan et al. reported lower or no toxicity of larger NPs, compared to smaller, even at higher concentration, which is likely related to their lower penetration through the cell membrane [34]. Higher toxicity of positively charged NPs compared with their same-sized negatively charged counterparts was also observed [35]. The cellular uptake of positively charged NPs is faster since it is driven by electrostatic attraction (cell membrane have slightly negative charge) [29]. Ligand density anchored onto surface of Au NPs is another important parameter altering their physical properties (e.g., hydrodynamic size, zeta potential, and mobility) and therefore influence interaction of Au NPs inside body, like cellular uptake or targeting specific cells [29,36].

This study deepens the information of our previous studies, investigating colloidal stability, structure, pH, viscosity, and density and type of chemical bond of PEGs ligands on Au NPs’ surface and their cytotoxic effect [37,38]. Colloidal Au dispersions were synthesized by cathode sputtering into liquid PEG substrates. Two types of PEG (i.e. PEG and PEG-NH_2_) were used as capturing media as well as stabilizing agents. Stability of PEGylated Au NPs was examined in terms of aging and thermal stress. Both Au NPs were subjected to antiviral assay. For possible impact of the prepared NPs on human health and environment, especially the potential for NP-induced toxicity, we have also studied biological response of our PEGylated Au NPs against human osteoblast and fibroblast cell lines.

## 2 Experimental section

### 2.1 Materials

PEGylated Au NPs were prepared by sputtering of gold into liquid medium. A starting material for synthesis of Au NPs was Au target (of 99.99 % purity, Safina, a. s., CZ). As for liquid media, which served as stabilizing agents as well, we used two types of polyethylene glycols (both supplied by Sigma-Aldrich Corp., US): (i) polyethylene glycol (PEG, *M*_w_ = 400 g/mol) and (ii) polyethylene glycol methyl ether amine (PEG-NH_2_, *M*_*w*_ = 500 g/mol). Amine terminated polyethylene glycol was mixed with pure PEG in weight ratio 20:1 (PEG:PEG-NH_2_).

### 2.2 Preparation of Au NP colloidal dispersions

Sputtering of Au into liquid media was performed on Sputter Coater SCD 050 (BalTec AG, CH) apparatus. The sputtering process was carried out at room temperature (RT ∼ 25 °C) under following conditions: working gas (Ar, purity 99.999 %, Siad a. s., CZ) pressure of 10 Pa, current of 30 mA, electrode distance of 5 cm. Au was sputtered into 2 mL of capturing media for 900 s. Then the mixture was diluted in distilled water in volume ratio of 1:9 (PEG:H_2_O). Viscosity of blank PEG solution and PEGylated Au NPs evaluated at 25 °C using Brookfield DV3T rheometer (AMETEK Inc., US; Small sample adapter with SC4-18 spindle) was 1.62 and 1.60 for PEG. Viscosity values of PEG-NH_2_ samples were 1.63 and 1.66 (blank and Au NPs, respectively). For the study of stability, the Au NP dispersions were thermally stressed in closed vial on magnetic stirrer with heating - Heidolph MR Hei-Tec (Heidolph Instruments GmbH & Co. KG, DE) at 37 or 100 °C or in fridge at 4 °C for 5 h. Au NPs stability was also examined after sterilization in 3870 M Large Capacity Manual Autoclave (Tuttnauer Corp., NL) at temperature of 121 °C for 23 min. Aging stability was investigated on Au NPs kept in closed vial at RT for 28 days. Detected results were compared with results of freshly prepared Au NPs (labelled as *fresh*).

### 2.3 Analytical apparatus

Size and shape of PEGylated Au NPs were investigated using transmission electron microscopy (TEM). Samples were prepared by depositing 8 μl of Au NP dispersion on carbon-coated copper grid (400 mesh) and an excess on the grid was removed by Whatman filtration paper. The samples were observed on TEM microscope JEOL JEM-1010 (JEOL Ltd., JP) operated at the accelerating voltage of 80 kV. Images were taken by CCD camera SIS Mega View III camera (Olympus Corp., JP) and analysed by AnalySIS v. 2.3 software (Münster, DE). Average size of the prepared NPs was determined using ImageJ software by measuring 150 particles.

Concentration of gold in colloidal dispersions was determined by atomic absorption spectroscopy (AAS) on spectrometer Agilent 280 FS AA (Agilent Technologies Inc., AU) with flame atomizer. For determination, absorption at wavelength of 242.8 nm was used. The error range of measurement was 0–4 %.

Differential ultraviolet–visible (UV–Vis) spectroscopy was used to study optical properties of colloidal Au NP dispersions. Absorption spectra were recorded on Lambda 25 spectrophotometer (PerkinElmer Inc., US) in spectral range 350–700 nm with a 1 nm data step and scan speed 240 nm/min. The colloidal dispersions were kept in 1 cm polystyrene cuvette. Reference spectrum of solvent (capturing medium) was subtracted from the measured spectrum of Au NP colloids.

Density of ligands present on NPs surface was determined using agarose gel electrophoresis on Horizon 11.14 gel electrophoresis apparatus (Biometra GmbH, DE). The mobility of variously functionalized PEGylated Au NPs was measured in 2 % solution of agarose gel in TBE buffer with constant voltage 60 V (8 V–1 cm of gel). The separation of particles took place for approximately 40 min.

pH of Au NPs in PEG and PEG-NH_2_, freshly prepared and thermally stressed, was determined by Orion Star A211 pH meter (Thermo Fisher Scientific Inc., US) at RT. The device was calibrated before measurement by Orion pH Buffer solutions at pH of 4.01, 7.00 and 10.01 (Thermo Fisher Scientific Inc., US). Relative accuracy of the device is ±0.002.

The chemical binding of variously terminated PEGs to the NPs’ surface was investigated by Fourier-transform infrared spectroscopy (FTIR). The analysis was performed on Nicolet iS10 FTIR spectrometer (Thermo Fisher Scientific Inc., US) with ZnSe ATR accessory. Data were measured in reflection mode and were detected by DTGS KBr detector. Spectra were recorded from 4000 to 650 cm^−1^ at resolution of 24 cm^−1^ with accumulation of 128 scans and Happ-Genzel apodization. Data were evaluated in Omnic™ Series Software (Thermo Fisher Scientific Inc., US). The peaks were fitted by Gaussian/Lorentzian function. Each system was measured twice and the spectra were averaged.

Zeta potential of freshly prepared and thermally stressed samples was measured using a Litesizer™ 500 (Anton Paar, Graz, Austria), with a light source of 658 nm red laser (40 mW) at 25 °C. The samples were placed in an Omega cuvette (∼300 μl per sample). Data were evaluated by accompanying software program, Kalliope™. Each sample was diluted in distilled water (in proportion 1:30) and sonicated for 15 min prior to zeta potential measurement and then measured three times. Zeta potential were also determined for Au NPs in cultivation medium (see below) used for cell cultivation (Au NP concentration 14 mg/L).

### 2.4 Antiviral assay

Antiviral effect of prepared AuNPs was tested against SARS-CoV-2 (strain hCoV-19/Czech Republic/NRL_6632_2/2020) propagated in Vero E6 (ECACC, Salisbury, UK) cell line using XTT cell proliferation assay. NPs dispersions were serially diluted (1:4), the starting concentration of NPs dispersions was 0.0116 mg/mL (AuNP_PEG) and 0.0119 mg/mL (AuNP_PEG-NH_2_), and added to Vero E6 cells (seeded in 96-well plate previous day in Dulbecco's Modified Eagle's Medium with 10 % fetal bovine serum, 100 U of penicillin/ml and 100 μg of streptomycin/ml [all Merck]). The plates were then incubated in a CO_2_ incubator at 37 °C for 1 h. Subsequently, 0.3 μl of viral suspension SARS-CoV-2 (multiplicity of infection = 0.02) per well was added and incubated for 72 h. Afterwards, 50 μl of XTT reagent (Sigma-Aldrich Corp., US) and electron coupling reagent (phenazine methosulfate [Sigma-Aldrich Corp., US]) was added and incubated for another 3–4 h. Cytopathic effect of each sample was determined from the absorbance measured at wavelength of 450 nm in EnVision plate reader (PerkinElmer Inc., US). Data were evaluated in GraphPad Prism software. Cytotoxic effect of PEGylated Au NPs against Vero E6 cell line was determined before the antiviral assay itself. Both cytotoxic and antiviral assay was performed in triplicate. Effect of Au NPs was compared with control samples (only PEG or PEG-NH_2_) and untreated control (cells without Au NPs and without both virus and Au NPs).

### 2.5 In vitro cellular tests

Human osteosarcoma cell line (SAOS-2, DSMZ GmbH, DE) and human fibroblast cell line (NHDF, Primary Dermal Fibroblast, Lonza Group AG, CH) were used for determination of PEGylated Au NPs effect on cell activity. Cells were seeded in 96-well plate (1.5∙10^4^ cell cm^−2^) and cultivated for 24 h in DMEM medium (Dulbecco's Modified Eagle's Medium, Gibco, UK) with addition of 10 % heat-inactivated fetal bovine serum (FBS, PAA Laboratories GmbH, AT), penicillin (20 μl/mL, Sigma-Aldrich Corp., US) and streptomycin (20 μl/mL, Sigma-Aldrich Corp., US) at 37 °C and in 5 % CO_2_ atmosphere in an incubator. Afterwards, three different concentrations of AuNPs in PEG and PEG-NH_2_ (diluted by cultivation medium to 1, 7 and 14 mg/L) were added to the cells for 24 h. Simultaneously, the cells were incubated with only functionalizing media (PEG or PEG-NH_2_, labelled CTRL) as control samples for only PEG effects. The effect of different treatments was determined using analysis of cell metabolic activity (Cell Titer 96 Aqueous One Solution Cell proliferation Assay, MTS, Promega Corp., US) performed according to the standard protocol. Absorbance was measured using multi-detection multi-plate reader (The Spark, Tecan Group Ltd., CH) at 490 nm and reference at 655 nm. The cell number was assessed by fluorometric quantification of DNA using CyQUANT Proliferation Assay Kit (Thermo Fisher Scientific, USA) according to the manufacturer's instructions. Fluorescence intensity at excitation ∼485nm/emission ∼530 nm was measured using Spark® multimode microplate reader (Tecan Group Ltd., CH) and statistically evaluated. Metabolic activity normalized to cell number (metabolic activity of single cell) was further normalized (in percentage) with respect to the cells cultivated without any treatment (untreated control cells - set as 100 %).

### 2.6 Statistics

The nonparametric Mann–Whitney *U* test was used to determine the significant differences between the datasets, and p values of less than 0.05 were considered to be statistically significant. Microsoft Excel 2016 (Redmond, Washington, USA), STATISTICA Software (StatSoft, Czech Republic) and the software GraphPad Prism 8 (GraphPad Prism Software Inc., San Diego, CA) were used to for graph generation and performing statistical analyses.

## 3 Results and discussion

The main goal of this study was to prepare stable PEGylated Au NPs by simple, reproducible and low-cost one-step approach of Au sputtering into pure PEG (AuNP_PEG) or PEG-NH_2_ (AuNP_PEG-NH_2_). Since prepared Au NPs can be potentially used for bio-applications, they were also investigated in terms of functionalizing ligands influence on human cells (possible cytotoxicity).

**Fig. 1:**
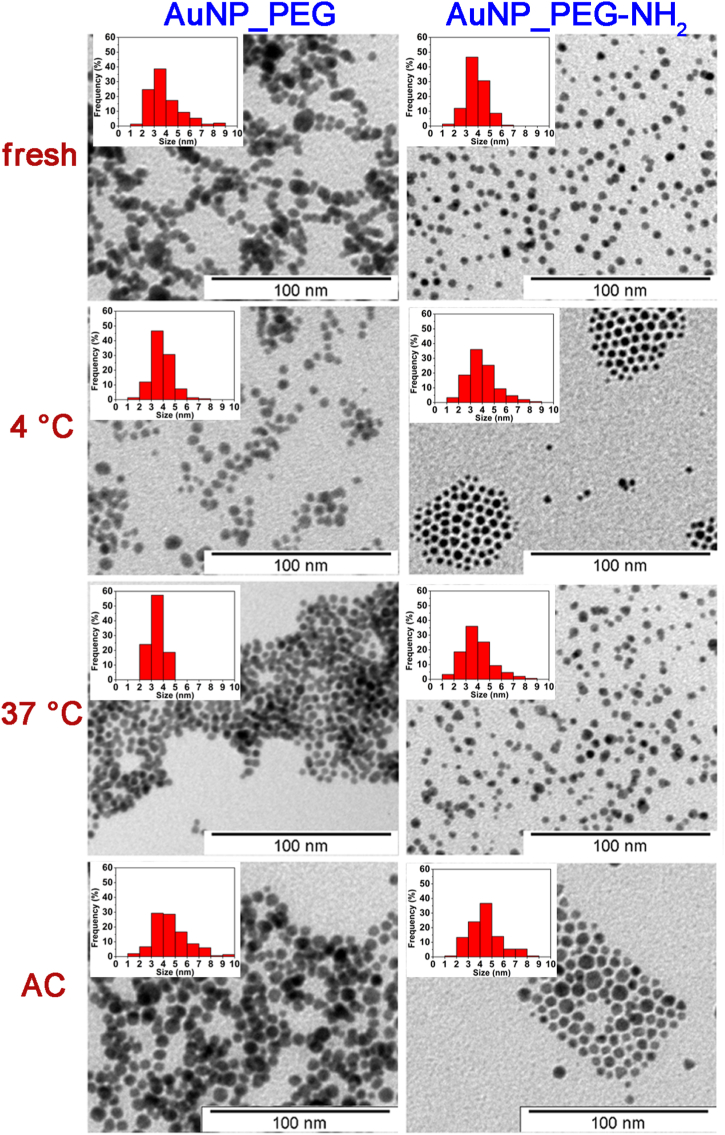
TEM images of PEGylated Au NPs freshly prepared, thermally stressed at 4 and 37 °C (for 5 h) and sterilized in an autoclave (121 °C for 23 min).

Fig. 1 documents TEM images of freshly prepared and thermally stressed Au NP dispersions. Au NPs were mostly of spherical shape in both used PEGs solutions and after all the tested treatments. AuNP_PEG tended to aggregate, which remained same after thermal stressing. AuNP_PEG-NH_2_ were homogenously spread but showed tendency to gather into groups after stressing at 4 °C and the autoclaving. TEM images were also used to determine the average size of Au NPs (see Table 1). The average diameter of both Au NPs was around 3.8 nm and increased by less than 1 nm after the sterilization.

**Table 1:** The average diameter (in nm) of PEGylated Au NPs, freshly prepared (RT), thermally stressed for 5 h at 4 and 37 °C and sterilized in an autoclave (AC) determined by TEM. Listed values and corresponding confidence intervals were calculated from measurement of 150 NPs.

Sample	Size (nm)			
	fresh	4 °C	37 °C	AC
**AuNP_PEG**	3.8 ± 0.3	3.8 ± 0.3	3.4 ± 0.1	4.6 ± 0.3
**AuNP_PEG-NH**_**2**_	3.8 ± 0.1	3.9 ± 0.2	3.7 ± 0.2	4.4 ± 0.2

The metal concentration in Au NP dispersions was determined by AAS analysis. Both Au NPs dispersions contained similar amount of Au (140 and 139 mg/L respectively). Changes of Au concentration caused by thermal stressing or aging are listed in Table 2 and Table 3, respectively. The Au content in both Au NPs dispersions was more or less same after the thermal stress, the slight deviation in determined values was within the error range of the measurement (4 %). After longer period (i.e. 28 days) on the bench, the content of Au slightly increased in AuNP_PEG (151 mg/L) and AuNP_PEG-NH_2_ (147 mg/L). However, the minor change may be also the result of manipulation with the Au colloids (sampling) rather than their instability. Overall, constant value of Au concentration confirms very good stability (temperature and time) of prepared colloids.

**Table 2:** Values of Au concentration (mg/L) in AuNP_PEG and AuNP_PEG-NH_2_ freshly prepared, thermally stressed for 5 h at 4 and 37 °C and sterilized in an autoclave (AC), examined by AAS. The error range of measurement was 0–4 %.

Sample	Au concentration (mg/L)			
	fresh	4 °C	37 °C	AC
**AuNP_PEG**	140	137	138	136
**AuNP_PEG-NH**_**2**_	139	134	134	135

**Table 3:** Changes of Au concentration (mg/L) in AuNP_PEG and AuNP_PEG-NH_2_ in the span of 28 days in RT, evaluated by AAS. The measurement error was 0–4 %.

Sample	Au concentration (mg/L)			
	fresh	7 days	14 days	28 days
**AuNP_PEG**	140	138	145	151
**AuNP_PEG-NH**_**2**_	139	143	148	147

**Fig. 2:**
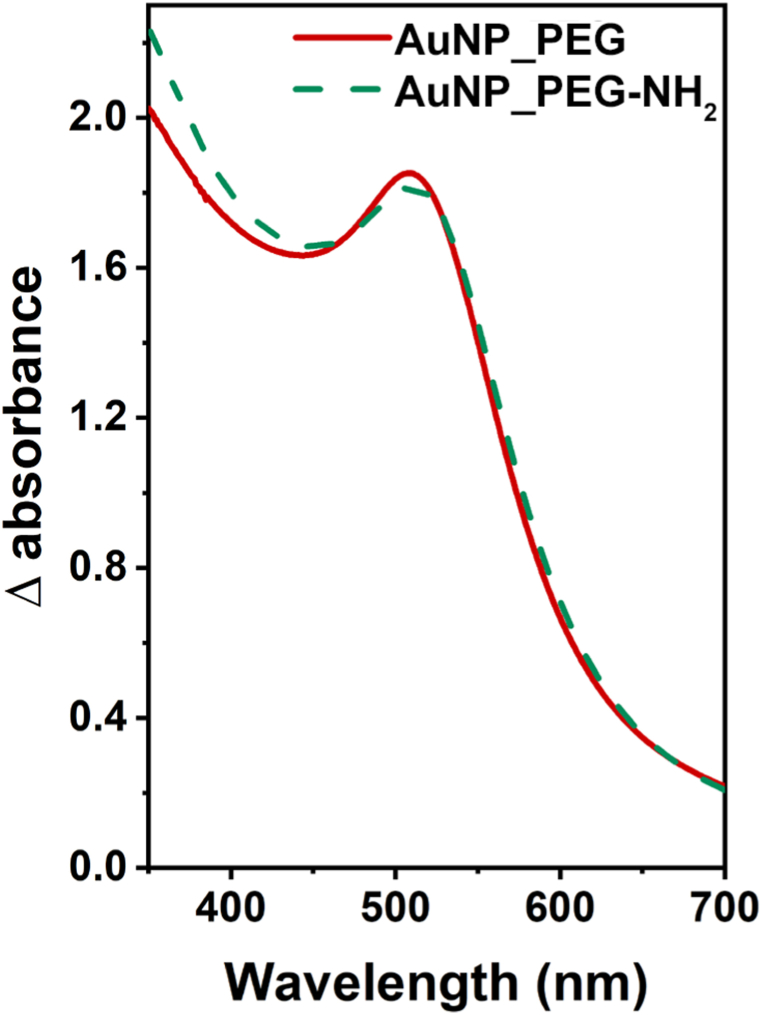
Differential UV–Vis absorption spectra of freshly prepared PEGylated Au NPs.

**Fig. 3:**
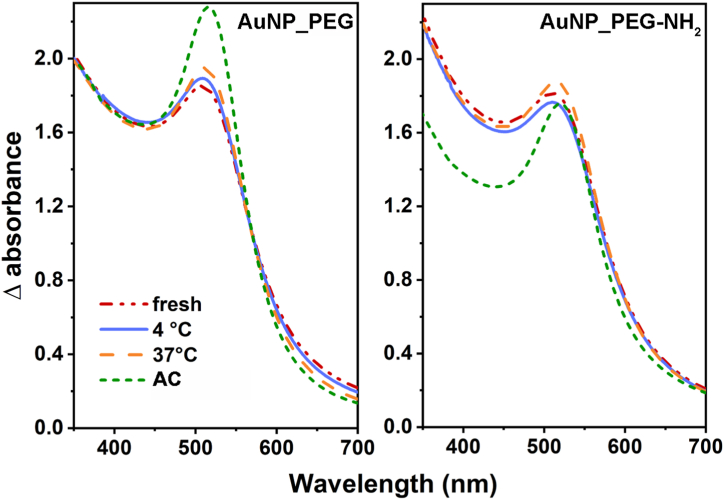
Thermal stability of AuNP_PEG and AuNP_PEG-NH_2_ stressed at 4 and 37 °C and sterilized in an autoclave determined by UV–Vis spectroscopy.

**Fig. 4:**
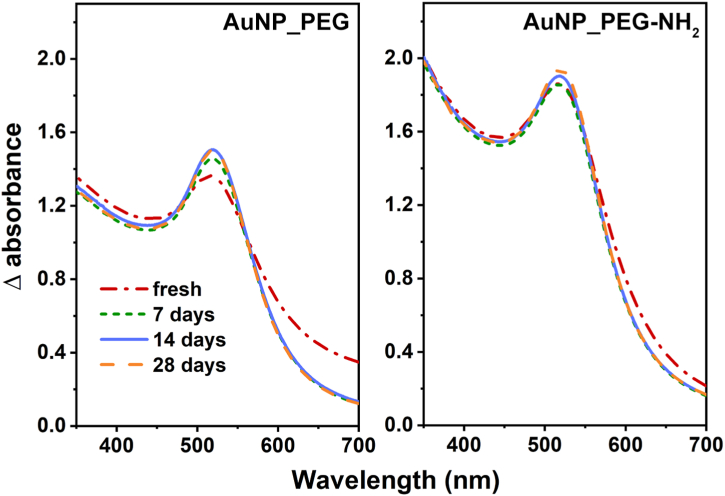
Aging stability of PEGylated Au NPs in the span of 28 days determined by UV–Vis spectroscopy.

Incident electromagnetic field (light) interacts with the conduction electrons of plasmonic NPs and causes their collective oscillation known as localized surface plasmon resonance (LSPR) [39]. The intensity and position of LSPR band characterize the size, the shape and the composition of the colloids, as well as the particles surrounding [[40], [41], [42][40], [41], [42]], therefore it can very well evaluate properties and stability of measured colloids. Differential UV–Vis spectra of the freshly prepared Au NP dispersions are compared in Fig. 2. Absorption spectra of both Au NPs are nearly identical, the AuNP_PEG having slightly higher absorbance due to higher concentration of Au NPs [43]. Both spectra show single LSPR band at wavelength of 509 nm, which confirms the presence of spherical Au NPs [21]. Fig. 3 compares differential UV–Vis absorption spectra of the freshly prepared Au NPs in different types of PEG with their thermally stressed counterparts. The most noticeable changes in Au NPs’ optical properties were caused by autoclaving, while thermal stressing at 4 and 37 °C induced slight to any difference. The absorption of AuNP_PEG slightly rose bit by bit with higher applied temperature: The autoclaving caused dramatic rise of absorption and slight redshift of LSPR band from 509 nm to 515 nm. The insignificant shift points to the good stability of prepared Au colloids. Higher absorption usually suggests an increase in NP concentration in dispersion [43]. Since the changes in Au concentration were minimal (see AAS results in Table 2), the absorbance is likely reflected by not only NPs parameters but by their surrounding environment and its dielectric properties or refractive index as well [44]. The higher absorption might be due to changes in the density of stabilizing agent creating NPs shell (in this case by PEG) [45]. The light absorption of AuNP_PEG-NH_2_ was similar after thermal stressing and even after autoclaving. During the stressing, however, SPR band gradually redshifted from 509 up to 520 nm. The redshift of LSPR band during thermal stressing, indicating increase in size of Au NPs [42,43,45], corresponds well with determined sizes of Au NPs (Table 1). The aging did not affect Au colloidal dispersion much, as can be seen in Fig. 4. The increase in absorbance of AuNP_PEG throughout the month is likely caused by binding of more PEG chains during the aging. The same can be observed in case of AuNP_PEG-NH_2_, although the changes are minimal. Overall, it can be stated that PEG-NH_2_ stabilize Au NPs better than PEG, even for a longer period. However with thermal stress, the stabilizing ability of PEG-NH_2_ significantly worsened compared to PEG [38].

**Fig. 5:**
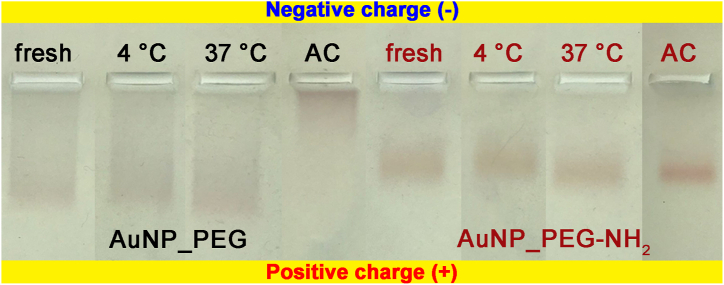
Image of gel electrophoresis of Au NPs stabilized by PEG and PEG-NH_2_, freshly prepared, thermally stressed at 4 and 37 °C and sterilized in autoclave.

Ligand density on NPs surface one of a key parameter for bioapplications [46]. Several works involving semiconductor quantum dots have shown that hydrodynamic radius, ligand density, and chain length influence NPs′ mobility [47]. Therefore, the density of each type of PEG functionalizing Au NPs surface was investigated by the gel electrophoresis. Fig. 5 documents the mobility of freshly prepared, thermally stressed and sterilized Au NPs functionalized by respective PEGs in agar gel. Au NPs moved in direction to positive electrode, indicating negative charge of the colloidal dispersions. The movement of AuNP_PEG was faster than AuNP_PEG-NH_2_. The lower mobility of AuNP_PEG-NH_2_ sample can be caused by (i) higher molar weight of PEG-NH_2_ than of PEG alone, (ii) higher density of stabilizing ligands on Au NP's surface (thicker softshell) or (iii) less negative charge of Au NPs (−15 mV for AuNP_PEG-NH_2_ and -20 mV for AuNP_PEG) [47,48]. However, the mobility of fresh and thermally stressed samples of the respective stabilizing agent were comparable. The most significant change was induced by sterilization. Sterilized AuNP_PEG moved much slower than AuNP_PEG-NH_2_. The reason may be thickening of PEG layer around NPs which can significantly hinder their mobility [47].

pH of Au NP dispersions is another parameter of great significance for bio-application. The pH of substances introduced into body should have similar value to the body to preserve the acid-base balance of biochemical reaction inside organism. Changes of pH values, caused by sputtering of Au into stabilizing agents and by applying of thermal stress, are documented in Table 4. The AuNP_PEG colloid were slightly acidic (pH around 6), after sterilization the pH value further decreased, likely due to increased dissolution of CO_2_ with higher temperature [49]. On the other hand, the pH of AuNP_PEG-NH_2_ solution were much more alkaline (pH over 10) owing to the presence of amine groups. The pH values slightly decreased with the higher applied temperature, again possibly in consequence of CO_2_ dissolution. Neither of prepared Au NP dispersion has pH comparable to blood pH (7,4) [50], thus they have to be adjusted for the potential bio-applications.

**Table 4:** pH values of freshly prepared and thermally stressed AuNP_PEG and AuNP_PEG-NH_2_. The relative accuracy of the device is ±0.002.

Sample	pH			
	fresh	4 °C	37 °C	AC
**AuNP_PEG**	5.7	6.2	5.8	4.1
**AuNP_PEG-NH**_**2**_	10.5	10.2	10.0	9.9

**Fig. 6:**
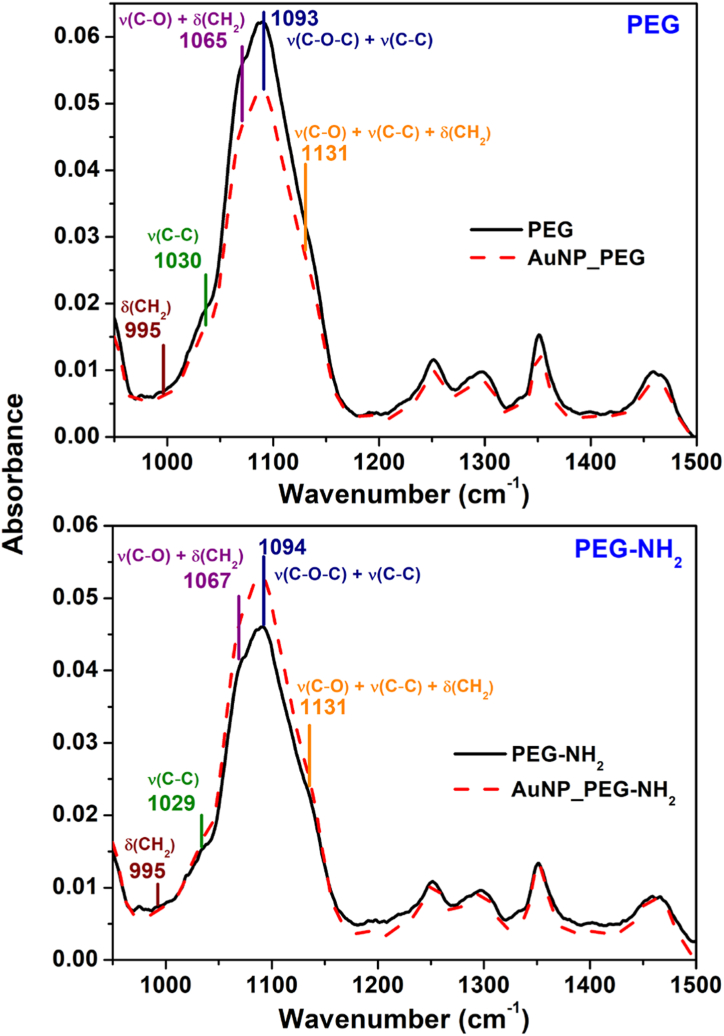
FTIR spectra of freshly prepared PEGylated Au NPs stabilized by (from the top to the bottom): PEG and PEG-NH_2_. Solid black line represents solutions of each PEG without Au NPs (CTRL) and red dash line Au NPs stabilized by given PEG. (For interpretation of the references to color in this figure legend, the reader is referred to the Web version of this article.)

**Fig. 7:**
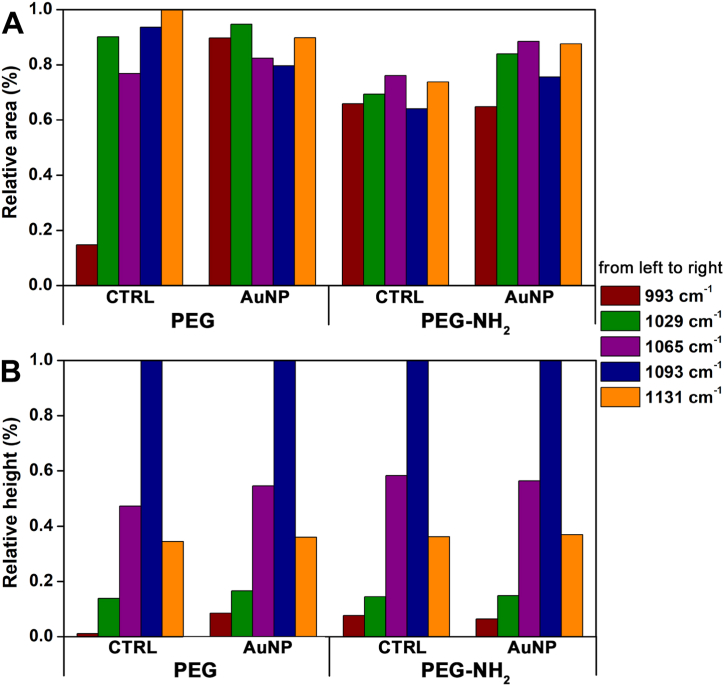
Relative area (A; in %) and relative height (B; in %) of the main of the chemical bands for PEG and PEG-NH_2_ determined by peak fit analysis from FTIR spectra. Left set of columns belongs to blank PEG solutions (CTRL). Wavenumber increases from left to right from 993 to 1131 cm^−1^.

The type of chemical bond that arises during the binding of PEG and PEG-NH_2_ onto Au NPs surface was investigated using FTIR by comparing spectra of PEGs only (CTRL) and corresponding Au colloid (Fig. 6). For better evaluation, the spectrum of water, which was obtained at the same conditions as samples spectra, was subtracted from the samplesˈ spectra. Minimal differences were observed on the first sight. For this reason, peak fitting analysis was created in the spectral region characteristic for C–O, C–C and C–H bending modes 1180-980 cm^−1^. The presented bands of various coupled modes are I) bending vibrations of CH_2_ (993 cm^−1^), II) stretching vibration of C–C (1029 cm^−1^), III) stretching vibration C–O coupled with rocking mode of CH_2_ (1065 cm^−1^), IV) stretching vibration of C–*O*–C with C–C stretching mode (1093 cm^−1^) and V) stretching vibration C–O coupled with stretching vibration of C–C coupled with rocking mode of CH_2_ (1131 cm^−1^). The assignment of the bands was based on Rozenberg et al. [51] and Vrandecic et al. [52] Fig. 7 documents relative area and relative height of these bands. The areas of fitting bands are related to the maximum of each band. Au NPs presence affects these bands. In the case of samples with hydroxyl group (see Fig. 7A – AuNP_PEG), the ratio between bands at 1065 and 1093 cm^−1^ (influence of CH_2_ and C–C, respectively) is changing. On the other hand, the ratio of these bands of the samples with amino group (Fig. 7A – AuNP_PEG-NH_2_) is almost the same. Therefore, it can be concluded that there is higher effect of Au NPs on vibration modes of PEG, while the effect of NPs in the presence of amino groups (PEG-NH_2_) was minimal. Relative heights of bands (Fig. 7 B) show the effect of Au NPs on the C–O, C–C and CH_2_ bands. The band at 1065 cm^−1^ increased at samples with hydroxyl groups and at samples with amino groups stayed almost unchanged. According to Rozenberg et al. this band was assigned to C–O mode [51]. The slight increase of intensity was observed at 1029 cm^−1^ assigned to C–C mode for all the systems. From these results, it can be supposed that the functional groups affect bonds with oxygen whereas nanoparticles affect skeletal C–C bonds.

**Fig. 8:**
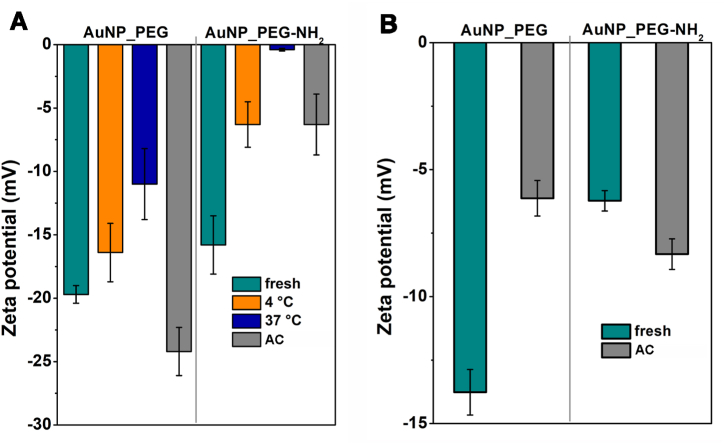
Zeta potential of Au NPs stabilized by PEG and PEG-NH_2_ determined in water (A) and in cultivation medium (B).

Zeta potential represents NPs surface charge that also suggest colloid stability and presence of functional groups on PEGylated Au NPs. The higher the zeta potential value is (either positive or negative), the less are NPs prone to aggregation [53,54]. Fig. 8 documents changes in zeta potential of the fresh and the thermally stressed Au NPs measured in water (Fig. 8A) and of the fresh and the autoclaved Au NPs in the cultivation medium (Fig. 8B). In water, zeta potential of all AuNP_PEG was significantly more negative than AuNP_PEG-NH_2_. Since the stability was good in both AuNP_PEG and AuNP_PEG-NH_2_ (see Fig. 3), the less negative charge is likely a result of –NH_2_ group presence on the surface of softshell [38,55]. That confirms FTIR spectroscopy outcome of PEG and PEG-NH_2_ grafting onto Au NPs surface through C–C skeletal bonds. Zeta potential of both AuNP_PEG and AuNP_PEG-NH_2_ shifted to less negative value with higher thermal stress, which is more pronounced in AuNP_PEG-NH_2_. More than instability, it can indicate the increasing thickness of softshell around NPs [47]. Sterilization in the autoclave shifted zeta potential to more negative values in case of AuNP_PEG. This shift may indicate that the PEG molecules grafted on the surface of NPs have undergone some level of degradation during the autoclaving process. Zeta potential of studied NPs in cultivation medium reached less negative values compared to the measurement in the water. The discrepancy is due to the higher salts content in the cultivation medium. The higher ionic strength results in the decrease of zeta potential. The different changes in zeta potential are also the result of the interaction between the salts or proteins in medium and the functional group of stabilizing PEG (-OH for PEG and –NH_2_ for PEG-NH_2_). Another parameter causing different shift of zeta potentials of Au NPs in both solution is different pH of NPs dispersions. The higher pH value, the more negative the zeta potential is [55,56]. Therefore it is expected that AuNP_PEG-NH_2_ would still have less negative, or even positive, surface charge at pH values (e.g. 5–6) in comparison with pH = 10 due to presence of amino-groups. The trend of zeta potential changes is similar at both NPs dispersions.

**Fig. 9:**
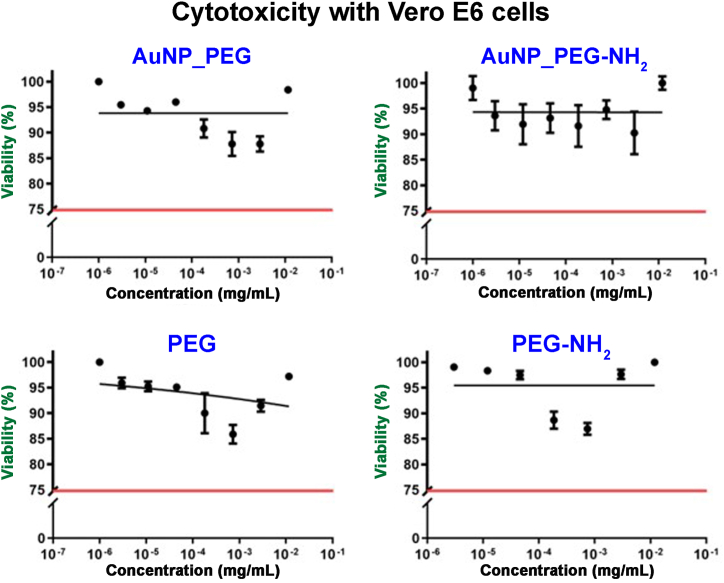
Cytotoxicity of PEGylated Au NPs and their corresponding PEG solutions to Vero E6 cells. Red line indicate cytotoxic level. (For interpretation of the references to color in this figure legend, the reader is referred to the Web version of this article.)

**Fig. 10:**
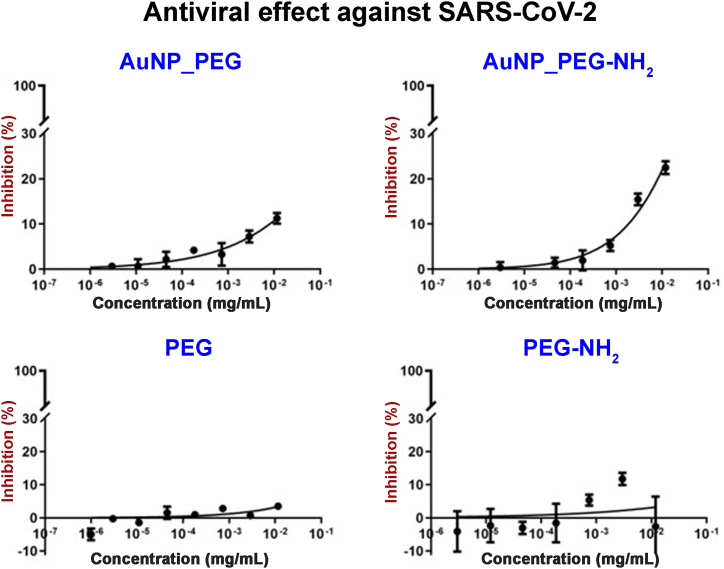
Antiviral effect of AuNP_PEG, AuNP_PEG-NH_2_ and their corresponding PEG solutions against SARS-CoV-2.

Antiviral effect of PEGylated Au NPs was investigated against SARS-CoV-2. Prior to antiviral assay, the Au NPs cytotoxicity to Vero E6 cells, which were used for virus propagation, was determined. Cytotoxic assay (see Fig. 9) indicated slight decrease of cells viability for both AuNP_PEG and AuNP_PEG-NH_2,_ as well as control samples (pure PEG or PEG-NH_2_). However, the decrease was not dramatic and according to Flahaut et al. [57], it is not a decrease to toxic level, which is stated as 75 % of untreated control (Fig. 9 – red line). Consequently, it was safe to proceed with antiviral assay. Inhibition of cytopathic effect of SARS-CoV-2 on Vero E6 cells is documented in Fig. 10. Unfortunately, only over 10–20 % of inhibition was observed for AuNP_PEG and AuNP_PEG-NH_2_, respectively. Therefore, prepared PEGylated Au NPs do not possess significant antiviral activity against SARS-CoV-2, however neither do they express cytotoxic effect on Vero E6 cell line.

Au NPs functionalized with different PEG ligands were also examined in terms of their effect (cytotoxic) on human fibroblast (NHDF) and human osteoblast cell line (SAOS-2). Both cell types were incubated with different particles – AuNP_PEG and AuNP_PEG-NH_2_ – of rising concentration for 24 h when their effect on cell number and metabolic activity of single cell was determined. The effect of the Au NPs sterilization in autoclave was also explored.

**Fig. 11:**
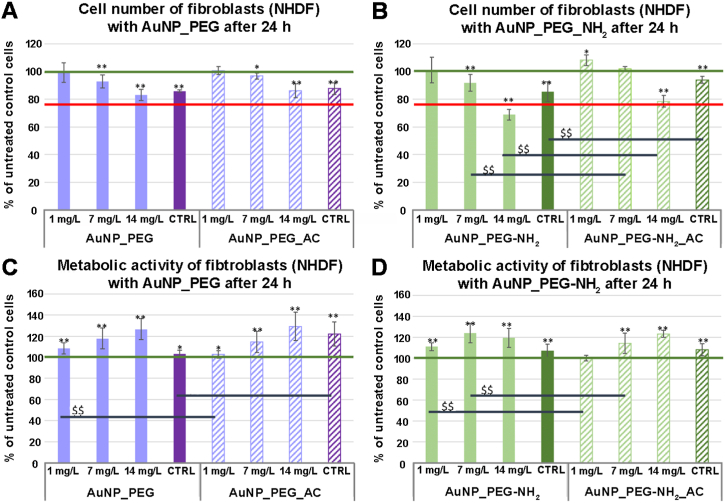
Cell number (A, B) and metabolic activity (C, D) of human fibroblasts (NHDF) after 24 h of incubation with AuNP-PEG and AuNP-PEG-NH_2_ (fresh – without a sign and after autoclaving – AC) and their corresponding blank PEG solutions (CTRL). Relative values are expressed as a percentage of untreated cells (100 %), ** represents significant difference to untreated control according to Mann-Whitney *U* test at p < 0.01; * represents significant difference to untreated control at p < 0.05. $$ represents significant difference between as prepared and after autoclaving samples. Green line – value of untreated cells, red line – cytotoxic value. (For interpretation of the references to color in this figure legend, the reader is referred to the Web version of this article.)

Fig. 11 A shows that cell number of fibroblasts was dose dependently decreased after incubation with AuNP-PEG, however, did not reach cytotoxic level. There was no difference in cell number of fibroblasts treated with the fresh and the autoclaved AuNP_PEG. The metabolic activity of remaining cells increased in dose-dependent manner suggesting reaction of cells on these NPs (Fig. 11C). On the other hand, AuNP_PEG-NH_2_ at the highest used concentration (14 mg/L) decreased fibroblast cell number to the cytotoxic level (Fig. 11B). In this case, a significant difference appeared between the fresh and the autoclaved samples as well. The greater reduction appeared in the fresh samples. The metabolic activity of single remaining fibroblast was significantly increased after incubation with both AuNP_PEG-NH_2_ samples, more with the fresh samples than the autoclaved ones (Fig. 11 D).

**Fig. 12:**
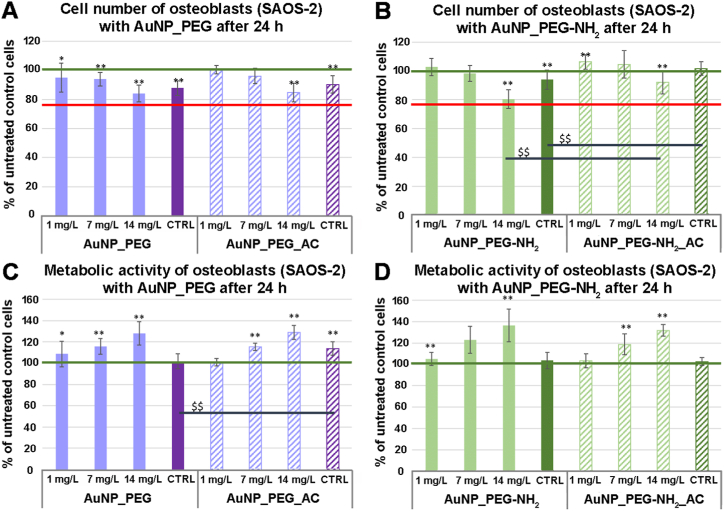
Cell number (A, B) and metabolic activity (C, D) of human osteoblasts (SAOS-2) after 24 h of incubation with AuNP_PEG and AuNP_PEG-NH_2_ (fresh – without a sign and after autoclaving – AC) and their corresponding blank PEG solutions (CTRL). Relative values are expressed as a percentage of untreated cells (100 %), ** represents significant difference to untreated control according to Mann-Whitney *U* test at p < 0.01; * represents significant difference to untreated control at p < 0.05. $$ represents significant difference between as prepared and after autoclaving samples. Green line – value of untreated cells, red line – cytotoxic value. (For interpretation of the references to color in this figure legend, the reader is referred to the Web version of this article.)

Similar effects of AuNPs occurred in human osteoblasts (Fig. 12), although less significant. However, Fig. 12 clearly shows dose dependent decrease in the cell number (Fig. 12 A, B) and corresponding dose dependent increase in the metabolic activity (Fig. 12 C, D). Related to these results, no difference in the effect of the fresh and the autoclaved Au NPs was observed.

To conclude, our PEGylated Au NPs have no cytotoxic effect on the tested human cells (fibroblasts and osteoblast) at even the highest tested concentration of Au NPs (irrespectively of PEG or PEG-NH_2_ modification). The sterilization of these Au NPs by autoclaving does not change their interaction with cells (revealing no differences in cell response), either, and thus can be used for bio-applications. That is also supported by results of Belinova et al. [58] showing their biocompatibility with immune cells (cell line and primary cells) as well as their mild immunomodulatory activity. These findings very well correspond with other studies confirming Au NPs minimal cytotoxic effect [59,60]. These results predispose our Au NPs to successful application in animal models and human medicine.

## 4 Conclusions

Widespread use of Au NPs in biological applications brings increasing requirements for their final properties. We have prepared PEGylated Au NPs by simple method of Au sputtering into pure or amine terminated PEGs. Afterwards, we have focused on stability and cytocompatibility of prepared the Au NPs, as those are one of the most important parameters for NPs potential applications. The possibility of their antiviral activity was also investigated. Prepared NPs were of spherical shape and size of approx. 3.8 nm (for both types of used PEG), determined by TEM analysis. Thermal stress did not affect NPs’ shape and size in all PEGs. Only after autoclaving, the size increased by less than 1 nm. Au concentration determined by AAS changed very slightly in both PEGs by aging or even by thermal stress. UV–Vis spectra of both PEG and PEG-NH_2_ stabilized Au NPs exhibited single LSPR band about wavelength of 500 nm, confirming presence of spherical Au NPs. UV–Vis spectra of their thermally stressed counterparts were more or less similar, except for autoclaved samples showing more pronounced changes. FTIR disclosed attachment of PEGs onto Au NPs through their skeletal C–C chain rather than the functional groups. According to gel electrophoresis data, the higher density of ligands can be assumed for AuNP_PEG-NH_2_. Unfortunately, no significant antiviral effect against SARS-CoV-2 was observed, however neither was cytotoxicity against Vero E6 cells used for virus propagation. Metabolic activity of both osteoblasts and fibroblasts also did not indicate any cytotoxic action of prepared Au NPs at neither of tested concentration. In conclusion, we have synthesized stable and non-cytotoxic PEGylated Au NPs even after autoclaving (sterilization). Therefore, our Au colloidal dispersions are safe for potential application in biomedicine especially in drug delivery, diagnostics and therapy.

## Data availability

Supplementary data to this article can be found online at DOI 10.5281/zenodo.11107817.

## CRediT authorship contribution statement

**Hoang Yen Nguyenova:** Writing – original draft, Visualization, Investigation, Formal analysis. **Marie Hubalek Kalbacova:** Writing – original draft, Formal analysis. **Marcela Dendisova:** Formal analysis. **Miriama Sikorova:** Formal analysis. **Jaroslava Jarolimkova:** Formal analysis. **Zdenka Kolska:** Resources, Formal analysis. **Lucie Ulrychova:** Investigation, Formal analysis. **Jan Weber:** Resources, Formal analysis. **Alena Reznickova:** Writing – review & editing, Validation, Supervision, Resources, Project administration, Methodology, Conceptualization.

## Declaration of competing interest

The authors declare that they have no known competing financial interests or personal relationships that could have appeared to influence the work reported in this paper.
